# A Smoking Cessation App for Nondaily Smokers (Version 2 of the Smiling Instead of Smoking App): Acceptability and Feasibility Study

**DOI:** 10.2196/29760

**Published:** 2021-11-17

**Authors:** Bettina B Hoeppner, Kaitlyn R Siegel, Hannah A Carlon, Christopher W Kahler, Elyse R Park, Susanne S Hoeppner

**Affiliations:** 1 Recovery Research Institute Department of Psychiatry Massachusetts General Hospital Boston, MA United States; 2 Department of Psychology University of New Mexico Albuquerque, NM United States; 3 Center for Alcohol and Addiction Studies Department of Behavioral and Social Sciences Brown University School of Public Health Providence, RI United States; 4 Health Promotion and Resiliency Research Intervention Program Psychiatry & Medicine Massachusetts General Hospital Boston, MA United States; 5 Center for Anxiety and Traumatic Stress Disorders Department of Psychiatry Massachusetts General Hospital Boston, MA United States

**Keywords:** nondaily, smoking cessation, smartphone app, positive psychology, mHealth, happiness, mobile phone

## Abstract

**Background:**

Recent evidence highlights the significant detrimental impact of nondaily smoking on health and its disproportionate prevalence in underserved populations; however, little work has been done to develop treatments specifically geared toward quitting nondaily smoking.

**Objective:**

This study aims to test the feasibility, acceptability, and conceptual underpinnings of version 2 of the *Smiling Instead of Smoking* (SiS2) smartphone app, which was developed specifically for nondaily smokers and uses a positive psychology approach.

**Methods:**

In a prospective, single-group study, nondaily smokers (N=100) were prescribed use of the SiS2 app for 7 weeks while undergoing a quit attempt. The app assigned daily positive psychology exercises and behavioral tasks every 2 to 3 days, which guided smokers through using the smoking cessation tools offered in the app. Participants answered surveys at baseline and at 2, 6, 12, and 24 weeks postquit. Feasibility was evaluated based on app use and acceptability based on survey responses. The underlying conceptual framework was tested by examining whether theorized within-person changes occurred from baseline to end of treatment on scales measuring self-efficacy, desire to smoke, and processing of self-relevant health information (ie, pros and cons of smoking, importance of the pros and cons of quitting, and motivation).

**Results:**

Participants used the SiS2 app on an average of 24.7 (SD 13.8) days out of the 49 prescribed days. At the end of treatment, most participants rated the functions of the app as *very easy to use* (eg, 70/95, 74% regarding cigarette log and 59/95, 62% regarding happiness exercises). The average score on the System Usability Scale was 79.8 (SD 17.3; *A* grade; *A+* ≥84.1, *B+* <78.8). Most participants reported that the app helped them in their quit attempt (83/95, 87%), and helped them stay positive while quitting (78/95, 82%). Large effects were found for within-person decreases in the desire to smoke (*b*=−1.5, 95% CI −1.9 to −1.1; *P*<.001; g_av_=1.01), the importance of the pros of smoking (*b*=-20.7, 95% CI −27.2 to −14.3; *P*<.001; g_av_=0.83), and perceived psychoactive benefits of smoking (*b*=−0.8, 95% CI −1.0 to −0.5; *P*<.001; g_av_=0.80). Medium effects were found for increases in self-efficacy for remaining abstinent when encountering internal (*b*=13.1, 95% CI 7.6 to 18.7; *P*<.001; g_av_=0.53) and external (*b*=11.2, 95% CI 6.1 to 16.1; *P*<.001; g_av_=0.49) smoking cues. Smaller effects, contrary to expectations, were found for decreases in motivation to quit smoking (*P*=.005) and the perceived importance of the pros of quitting (*P*=.009). Self-reported 30-day point prevalence abstinence rates were 40%, 56%, and 56% at 6, 12, and 24 weeks after the quit day, respectively.

**Conclusions:**

The SiS2 app was feasible and acceptable, showed promising changes in constructs relevant to smoking cessation, and had high self-reported quit rates by nondaily smokers. The SiS2 app warrants testing in a randomized controlled trial.

## Introduction

### Background

Nondaily smoking continues to be a public health issue with limited empirically supported options to support smoking cessation. Currently, 25.4% of all adult smokers are nondaily smokers [1]. This prevalence has been increasing over the past decade, from a prevalence of 20.2% in 2008 [2] to 25.4% in 2018 [1]. In 2007, the National Institutes of Health highlighted nondaily smoking as a public health issue [3]. Now, more than a decade later, there continues to be a lack of smoking cessation support for this important population of smokers.

For most nondaily smokers, nondaily smoking is a long-term pattern of smoking, not just a transitional phase [4]. Prior research has established that nondaily smokers are more motivated to quit smoking than daily smokers [5]. Moreover, a growing body of research has documented that nondaily smokers differ from daily smokers in terms of numerous characteristics relevant to the process of quitting smoking, including smoking motives [6] and situational antecedents of smoking [7]. These facts argue for the development of targeted and tailored public health efforts to support nondaily smokers in quitting smoking.

Newer emerging evidence has highlighted the urgency of addressing nondaily smoking. Contrary to initial beliefs, the adverse health impact of nondaily smoking is not negligible. Recent research has shown that the mortality risk of native nondaily smokers (ie, those who have never smoked on a daily basis) compared with never smokers is 72% higher [8]. Impacts on all-cause mortality risk have been observed for smoking as few as 6-10 cigarettes per month [9]. These findings clarify that nondaily smoking has a substantial detrimental impact on health. In addition, vulnerable and underserved populations, among whom nondaily smoking is particularly prevalent, are disproportionally affected. These populations include racial and ethnic minority groups [10-12] and persons with mental health and substance use challenges [13]. Thus, addressing nondaily smoking is also a matter of health equity.

To date, very little research has been conducted to develop smoking cessation support approaches geared specifically toward nondaily smokers. Large randomized controlled trials of nicotine replacement therapy (NRT) for nondaily smokers failed to show efficacy in achieving smoking abstinence in comparison with placebo or counseling-only conditions [14,15]. Of note, in these trials, participant adherence to NRT was lower than recommended by study staff, suggesting a lack of interest among nondaily smokers in NRT as a smoking cessation aid. This observation is in line with other studies that showed that, among college students, nondaily smokers were less likely to be interested in using pharmacotherapy than daily smokers [16] and that nondaily smokers did not view nicotine addiction as relevant to their efforts to quit [17]. This points to the importance of behavioral approaches for nondaily smokers, where mobile technology can play a critical role.

Recent years have seen an uptake in the use [18,19] and clinical linkage of mobile technologies to support smoking cessation, where it is now recommended that health care professionals connect their smoking patients to mobile health (mHealth) resources for smoking cessation [20]. Smartphone app technology may be particularly useful for nondaily smokers, who are less likely to engage in traditional smoking cessation interventions [16]. Beyond treatment modality, the content of intervention approaches for nondaily smokers needs to be tailored to address the unique characteristics of nondaily smoking, as constructs most relevant to smoking cessation for daily smokers appear to be less salient to nondaily smokers as they prepare for the quit day [17].

### Development of the Smiling Instead of Smoking App

To address these needs, we developed a smoking cessation app called *Smiling Instead of Smoking* (SiS) [21]. This app builds on the development of positive psychotherapy for smoking cessation [22,23] and adapted its core principles to the app environment. Our rationale for choosing this approach was based on prior research, which indicated that nondaily smokers did not view nicotine addiction as relevant to their efforts to quit but instead viewed themes related to a positive self-identity and wellness as more important [17]. The app’s overall conceptual model, as described elsewhere [21,24], is based on social cognitive theory, [25] where goal setting, self-efficacy, and knowledge are leveraged to support behavioral change. Within this framework, the SiS app uses a positive psychology approach to achieve 2 goals: (1) to foster engagement with the app and its smoking cessation content and (2) to support positive affect while smokers undertake the quit attempt. Regarding engagement, positive psychology interventions for health behavior change have been found to be highly appealing to patients [26], resulting in better treatment adherence [27,28] and engagement [29]. Regarding the utility of high positive affect during smoking cessation, findings from laboratory and real time studies have shown that high positive affect is associated with increased self-efficacy [30], decreased desire to smoke [31,32], and greater readiness to process self-relevant health information [33], all of which are constructs highlighted in dominant theories of health behavior change as playing a causal role in the process of behavioral change, including in the health belief model [34], social cognitive theory [35], the theory of planned behavior [36], the transtheoretical model [37], and the relapse prevention model [38].

We tested the feasibility and acceptability of version 1 of this app (SiS1) in an initial sample of 30 nondaily smokers [24]. Initial testing of this app showed excellent app use, favorable ease of use and usefulness ratings, and significant within-person changes in line with our conceptual model. Self-reported abstinence rates were also fairly high (53% self-reported abstinence 6 months after quitting) [24].

**Figure 1:**
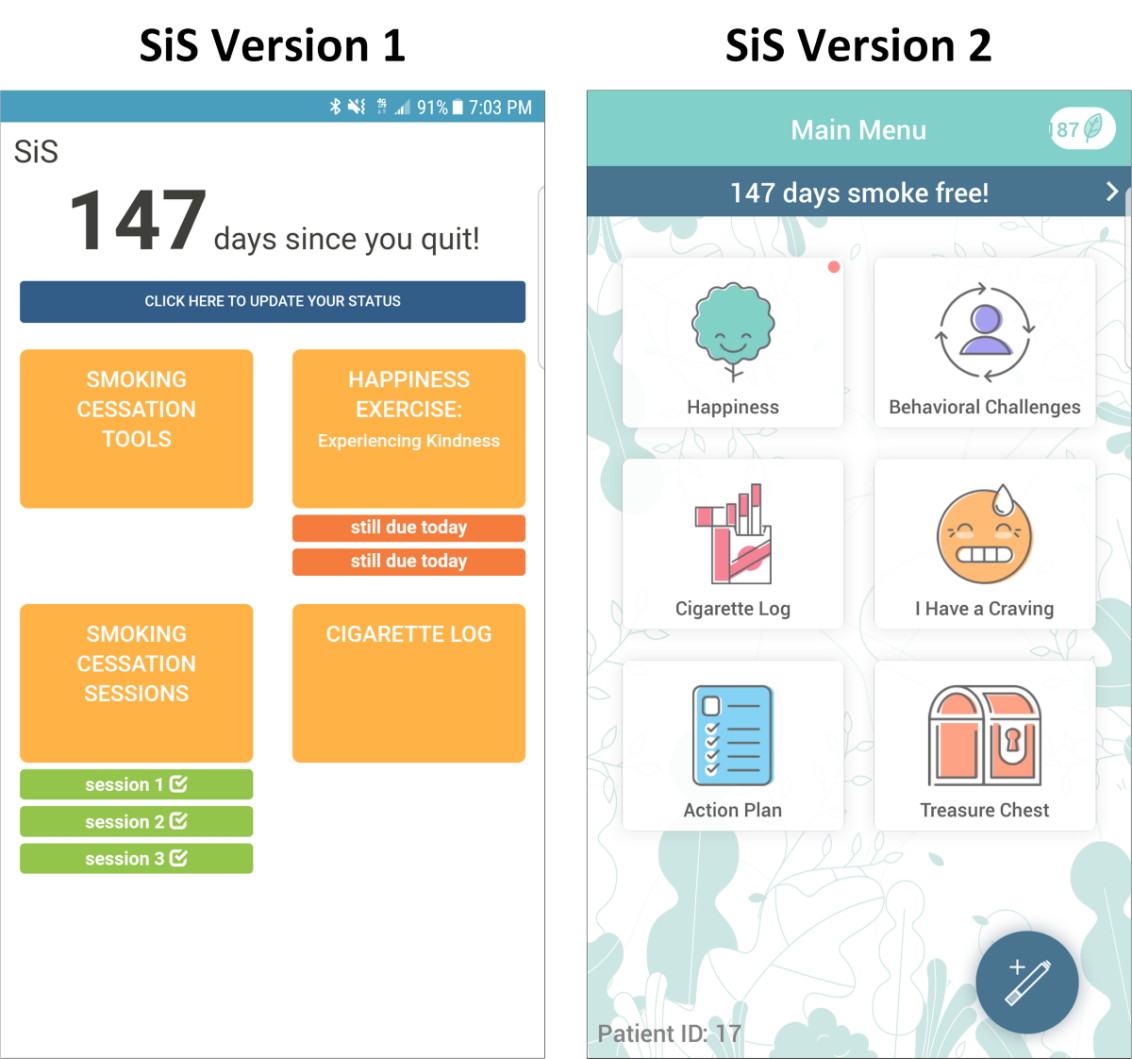
Main menu of Smiling Instead of Smoking app versions 1 and 2. SiS: Smiling Instead of Smoking.

The SiS2 app was developed based on the results of and feedback from participants using the SiS1 app [24]. In going from the SiS1 to the SiS2 app, we made some overall stylistic changes, such as redesigning the user interface (Figure 1), expanding the variety of daily positive psychology exercises, and adding gamification components (ie, points for completing tasks and a distraction game called *Magma Bear*).

**Figure 2:**
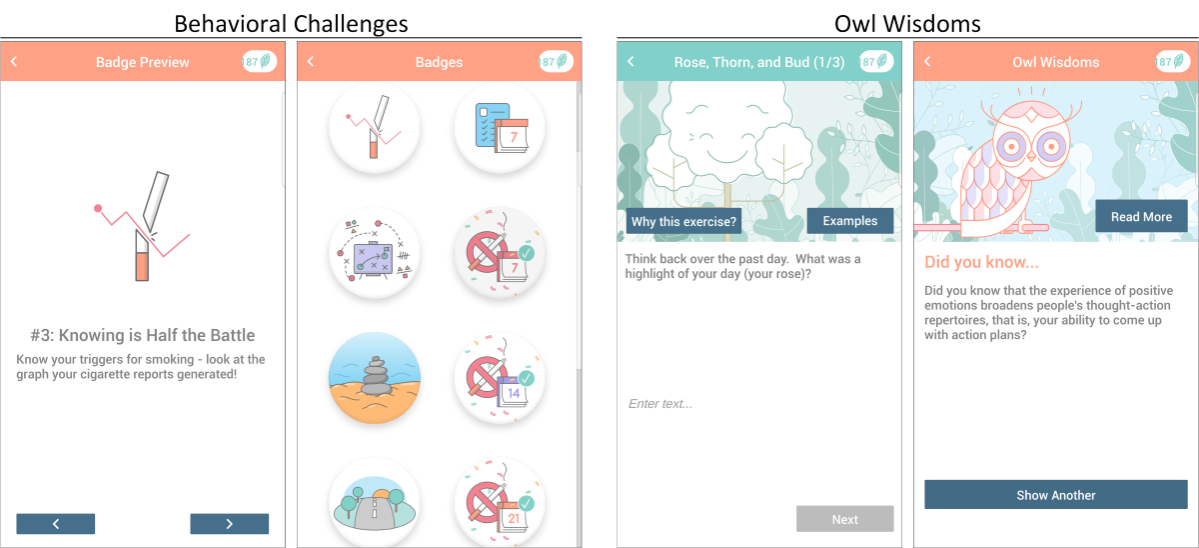
In going from Smiling Instead of Smoking app version 1 to version 2, behavioral challenges (A) were added to engage app users with the smoking cessation tools and (B) information was added to explain the positive psychology approach via "read more" buttons and Owl Wisdoms.

We also made 2 targeted changes to address specific weaknesses. We wanted to make the smoking cessation content more accessible, and we wanted SiS app users to better understand why our positive psychology approach may be useful. To accomplish the former, we replaced our weekly, wordy smoking cessation tutorials that were part of SiS1 with frequent, brief *behavioral challenges* in SiS2. With regard to affecting perceptions about the usefulness of positive psychology for smoking cessation, we added quite a bit of information on this topic throughout the app. This informational content was optional, and we conceptualized it as an invitation to learn more. To draw attention to this information, SiS2 used a new *Owl Wisdom* mechanism, where the app sent push notifications to app users every 3-4 days to share with them a relevant scientific finding related to happiness or positive affect and its connections to smoking outcomes, health, and well-being (Figure 2).

In study 2, we also changed important parameters regarding the *ask* from participants; that is, instead of onboarding participants in person, as we had done in study 1, we onboarded participants remotely via phone calls in study 2. We also increased the length of the *prescribed* app use from 3 weeks (study 1) to 7 weeks (study 2), as study 1 participants had asked for longer app support. We evaluated the acceptability and feasibility of this new approach in the same manner as in study 1.

### Objective

In this paper, we report the outcomes of a study (NCT03951766) examining version 2 of the SiS app (SiS2). Our goals are (1) to assess the feasibility and acceptability of the app as measured by actual app use and ratings of usefulness and ease of use, (2) to test whether within-person changes in line with the underlying conceptual model are observable, and (3) to report smoking cessation outcomes. Regarding the within-person changes, we hypothesize that participants in this study would show, by the end of treatment, an increased self-efficacy to abstain from smoking, reduced desire to smoke, and more positive processing of self-relevant smoking information (eg, decreased perceived pros of smoking and increased perceived cons of smoking). In line with our conceptual model, we do not hypothesize that positive affect would increase during this trial. Rather, the app’s goal is to maintain a positive affect while smokers undergo a quit attempt.

## Methods

### Participants

Participants were adult nondaily smokers who were interested in using a smartphone app to help them quit smoking (recruited from June 11, 2019 to November 15, 2019). Study recruitment information was displayed on Craigslist, Facebook, Reddit, Smokefree.gov, ClinicalTrials.gov, a study recruitment website at the Massachusetts General Hospital, our study website, and websites of local universities. Recruitment was bolstered through word-of-mouth referrals by participants in our first pilot study and the study staff. To participate, participants had to be aged >18 years, be current nondaily smokers who smoked at least weekly but <25 out of the past 30 days, own an Android or iPhone smartphone, be willing to make a quit attempt as part of the study, be willing to name friends or family members who could help study staff by updating contact information for follow-up assessments, and be fluent in English. The study (NCT03951766) was conducted entirely remotely and was approved by the Mass General Brigham institutional review board. All participants provided informed consent.

### Procedure

Interested participants were phone-screened and asked to complete a screening test. To pass, participants had to complete a web-based survey and correctly respond to 5 haphazardly placed check questions that tested if respondents were reading survey items. Participants who passed the screening survey were then asked to provide contact information for 2 collaterals who would be able to assist research staff in locating participants for follow-ups, if necessary. Participants were notified by phone if they were eligible, and during this phone call, study staff asked what participants had chosen as their designated quit day to set up the app onboarding call. During the onboarding call, which also served as the study’s enrollment call, study staff verified participants’ nondaily smoking status and then guided participants through downloading, installing, and using the app. This phone conversation focused on the app and how participants should be using it during the prescribed treatment period. Beyond walking participants through the app, the study staff did not offer further smoking cessation advice. The recording of their app data was confirmed by the study staff during this call. Participants were then asked to use the app for a period of 7 weeks (1 week prequit and 6 weeks postquit) and to complete follow-up surveys on the web 2, 6, 12, and 24 weeks after their initially chosen quit day. Participants received US $25 for completed surveys or US $10 for incomplete surveys or surveys with failed check items. They received US $50 for the week 6 survey (end of treatment), which was longer than the other surveys. Participants provided their social security numbers to enable remuneration by check. All surveys were administered via the electronic data capture system REDCap (Research Electronic Data Capture) [39].

**Figure 3:**
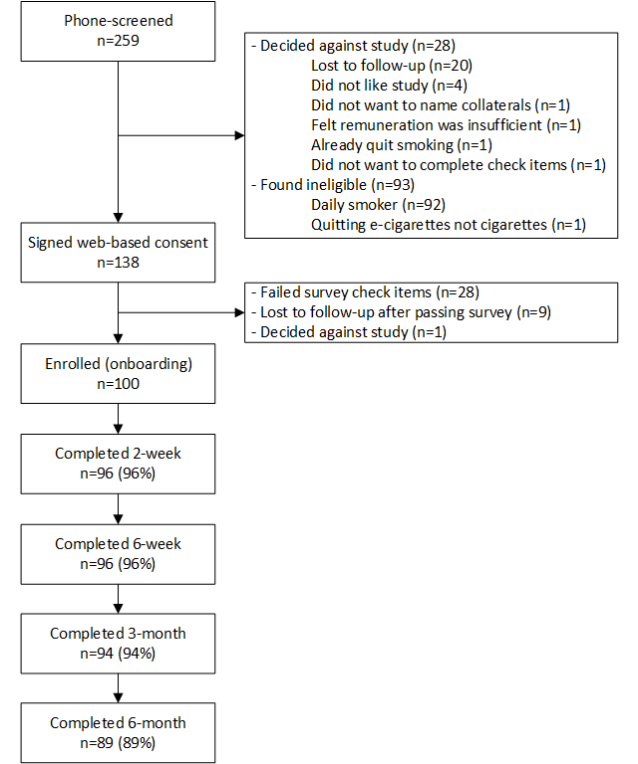
Consolidated Standards of Reporting Trials diagram for Smiling Instead of Smoking app study 2.

In total, we phone-screened 259 individuals (Figure 3). Of these 259 individuals, 93 (35.9%) were not eligible (of the 93 individuals, 92 [98.9%] were not eligible as they were daily smokers and 1 [1.1%] were not eligible as they wanted to quit vaping), and 28 (10.8%) decided against the study. The remaining 53.5% (138/259) signed the web-based consent and started the screening test. Of these 138 individuals, 28 (20.3%) failed the check items embedded in the survey, and 10 (7.2%) passed but decided against the study at this point. The remaining 72.5% (100/138) of individuals were onboarded to SiS2.

Participants who missed scheduled assessments were contacted with multiple reminders and were contacted for subsequent assessments unless they actively withdrew from the study. Survey completion rates were 96%, 96%, 94%, and 89% for follow-up surveys 2, 6, 12, and 24 weeks after the chosen smoking quit date, respectively (N=100). For half of the participants (49/100, 49%), the 24-week follow-up occurred after the onset of the COVID-19 pandemic in the United States.

### Treatment

Participants received the SiS2 app, which was based on existing, empirically tested positive psychology exercises [40-44], published guidelines for smoking cessation (ie, US Clinical Practice Guidelines) [45], and user feedback from nondaily smokers who used version 1 of the app [24]. The app engaged participants in daily positive psychology exercises over the course of 7 weeks, as well as temporally appropriate *behavioral challenges* every 2-3 days that were designed to engage participants with ad libitum tools offered by the app. The SiS2 app used 2 new happiness exercises in addition to the 3 exercises used in the SiS1 app (ie, *3 Good Things*, where participants entered text describing 3 good things that happened to them that day; *Savoring*, where participants entered text describing 2 experiences they savored, and *Experiencing Kindness*, where participants described an act of kindness they did or one they witnessed). The new exercises were *Rose, Thorn, and Bud*, where participants described one good and one challenging thing in the past 24 hours and one thing they looked forward to in the next 24 hours, and *Reliving Happy Moments*, where participants viewed and then described a picture they had taken that made them happy [41]. One of these five exercises was chosen at random each day by the app to be completed that day. The ad libitum tools offered tracking functionality (ie, logging smoked cigarettes), graphical summaries (ie, pie chart of reasons smoked with matching strategies to stay smoke free in such situations), reminders (ie, a tool to send push notifications to stay smoke free at specific times), note keeping (ie, personal reasons for quitting), and information (eg, benefits of quitting and strategies). The SiS2 app used push notifications in a variety of ways to engage app users (eg, reminders for missed exercises and push notification for new *behavioral challenges* and *Owl Wisdoms*). The design of the SiS2 app is evidence based and in line with all 6 published recommendations for smoking cessation apps [46]: it (1) is available at no cost, (2) keeps information private (ie, saved on our hospital servers), (3) matches individual needs and interests (eg, suggested strategies are matched to logged smoking reasons), (4) adapts as one’s needs and interests change (eg, behavioral challenges are anchored on the resettable quit day to be temporally appropriate), (5) helps to manage nicotine withdrawal symptoms (eg, specific strategies are offered in the app), and (6) allows users to track progress (eg, keeps track of days since quit day, provides graphs over time, and awards badges for achieving milestones).

### Measures

#### Baseline Characteristics

During the web-based baseline survey, sociodemographic information was collected, including age, gender, race, and education level. Participants also answered questions about their smoking characteristics (ie, number of days smoked in the past 30 days, number of cigarettes smoked per smoking day, history of daily smoking—yes or no—and past quit attempts).

#### Measures of Feasibility and Acceptability

##### App Use

App use was automatically recorded by the app, which time stamped every interaction with the app. From these data, we calculated the number of days on which participants used the app during the *prescribed* period of app use (ie, 0-49 days) and examined the number of days per week participants used the SiS2 app.

##### System Usability Scale

App usability was measured at the end of treatment using the 10-item System Usability Scale (SUS) [47], where we used the phrase *SiS app* in place of *this system*. Participants rated items (eg, “I thought the SiS app was easy to use.”*)* on a 5-point Likert scale (1=strongly disagree to 5=strongly agree). Scores are interpreted as *grades*: *A+* for scores ≥84.1, *A* for scores 84.1-78.8, *B+* for scores <78.8; scores <70 indicate *below average* usability [48].

##### App’s Ease of Use and Usefulness

At the end of treatment (week 6), participants rated the ease of use and usefulness of each component of the app (17 items each, listed in the *Results* section) on a 4-point Likert scale, where ease of use was rated as 0=not easy at all, 1=somewhat easy to use, 2=easy to use, or 3=very easy to use, and usefulness was rated as 0=not at all useful, 1=somewhat useful, 2=useful, or 3=very useful.

##### Perceptions of How the App Might Have Helped

At the end of treatment (week 6), participants provided their level of agreement (5-point Likert scale, 1=strongly disagree to 5=strongly agree) on 17 items pertaining to the helpfulness of the app during the quitting process (eg, in preparing for the quit attempt and during risky situations). Participants also indicated whether the app helped them in their quit attempt and if they would recommend it to a friend who wanted to quit smoking (yes or no).

#### Measures of Constructs Hypothesized to Change Over Time

As part of all surveys, participants completed the following scales:

##### Self-Efficacy

The Smoking Self-Efficacy Questionnaire (12-items; slider scale; 0=not at all confident to 100=extremely confident) [49] assesses confidence to abstain from smoking when faced with internal stimuli (eg, “when I feel very anxious”) and external stimuli (eg, “after a meal”).

##### Desire to Smoke

The Brief Questionnaire of Smoking Urges [50] uses 10 items on a 7-point Likert scale (1=strongly disagree to 7=strongly agree) to measure one’s desire to smoke as it relates to reward (eg, “A cigarette would taste good now”) and relief from negative affect (eg, *“*I could control things better right now if I could smoke”).

##### Processing Self-Relevating Health Information

The Attitudes Towards Smoking Scale [51] assesses participants’ feelings toward adverse effects, psychoactive benefits, and pleasures of smoking. Participants rated 18-items (eg, “smoking is ruining my health”) on a 5-point Likert scale (1=strongly disagree to 5=strongly agree). The Decisional Balance Inventory for Smoking–Short Form (6-items; slider scale; 0=not at all important to 100=extremely important) [52] assesses how expectations that are positive (eg, “Smoking cigarettes relieves tension.”) and negative (eg, “I’m embarrassed to have to smoke*”*) weigh in on one’s decision to smoke at that moment (ie, “right now”). The impact of perceived benefits and barriers on quitting smoking was evaluated using 2 single-item measures: (1) “Think about all the things you LIKE/LOVE about quitting/being smoke-free; taken together, how important are those things to you RIGHT NOW?” and (2) “Think about all the things you DISLIKE/HATE about quitting/being smoke-free; taken together, how important are those things to you RIGHT NOW?”; both were rated on slider scales ranging from 0=not at all to 100=extremely important*.* The Commitment to Quitting Smoking Scale [53] asks participants to rate their level of agreement (Likert scale, 1=strongly disagree to 5=strongly agree) on 8-items assessing motivation to quit smoking (eg, “I’m not going to let anything get in the way of my quitting smoking”). In addition, a single-item slider scale (0=not at all to 100=extremely motivated) directly asked participants, “How MOTIVATED are you to quit smoking/stay quit?*”*

##### Positive Affect

The Positive and Negative Affect Schedule [54] uses 20 items (10 positive adjectives and 10 negative adjectives) on a 5-point Likert scale (1=very slightly or not at all to 5=extremely) to assess the extent to which participants experienced positive and negative affect in the past week. Participants also answered 2 questions using a single-item slider (0=not at all to 100=extremely) to indicate their happiness in the moment and over the past week*.* Overall life-satisfaction and happiness were assessed using the Satisfaction with Life Scale (5 items; Likert scale ratings from 1=strongly disagree to 7=strongly agree) [55] and the Subjective Happiness Scale (4 items; item-specific anchor points; eg, for “Compared with most of my peers, I consider myself [...],” ratings range from 1=less happy to 7=more happy) [56]*.*

#### Exploratory Outcome—Self-reported Abstinence

During each survey, participants were asked to rate their smoking status using the following options: “I smoke daily,” “I smoke nondaily (and have smoked in the past 7 days),” “I smoke nondaily (but have NOT smoked in the past 7 days,” and “I do not smoke at all.” Participants who reported not smoking at all were then asked if they had been completely abstinent since their originally chosen quit day (if no, then since when), during the past 7 days, and during the past 30 days.

We did not perform biochemical tests to confirm self-reported abstinence in line with guidance (at that time) that such tests should not be used for studies with no face-to-face contact and studies in which data are optimally collected through the internet, telephone, or mail [57].

### Analytic Strategy

We used SAS 9.4 for all analyses. To describe feasibility, acceptability, and smoking cessation outcomes, we calculated descriptive statistics. To test if theorized within-person changes occurred from baseline to end of treatment, we fit one repeated measures mixed effects model per construct hypothesized to change over time (ie, self-efficacy, desire to smoke, and processing of self-relevant health information). In these models, the sole predictor was time, called TIME, modeled categorically (ie, baseline, 2, 6, 12, and 24 weeks). Observations were modeled as nested within individuals using an unstructured covariance matrix. Per protocol, the primary end point of interest was end of treatment (ie, 6 weeks after the chosen quit date). Thus, using this model, we reported the pairwise contrast between baseline and week 6. Given the exploratory nature of this study, we did not correct for multiple testing. Effect sizes for within-person changes from baseline to week 6 in each outcome measure were reported as Hedges g_av_, a bias-corrected effect size estimate recommended for correlated samples [58]. We used the same modeling approach to capture changes in positive affect. For the 2 outcomes that indicated significant effects counter to the hypothesized direction (ie, motivation to quit and the perceived importance of the pros of quitting), we used post hoc analyses to see whether changes from baseline to end of treatment differed between participants who achieved 30-day point prevalence abstinence at the end of treatment versus those who did not. In these post hoc models, we added quit status as a binary predictor (QUIT: 1=abstinent, 0=not) and its interaction with TIME to the model. We interpreted a significant QUIT*TIME effect as an evidence of differential changes over time based on quit status.

## Results

### Participant Characteristics

Participants were predominantly (70/100, 70%) converted nondaily smokers (ie, people who had smoked daily previously), who smoked an average of 4.6 (SD 3.3) cigarettes per smoking day on 14.7 (SD 4.6) days out of the past 30 days, in line with expected rates of smoking in nondaily smokers [59]. Many had made a previous quit attempt, also in line with expectations for this type of smoker [5]. Demographics are reported in Table 1.

**Table 1:** Demographics and smoking characteristics (N=100).

Characteristics				Values
**Demographics**				
	Age (years), mean (SD)			35.9 (11.4)
	Gender (female), n (%)			61 (61)
	**Race, n (%)**			
		White	75 (75)	
		Black	14 (14)	
		Other or unknown	11 (11)	
		Hispanic	12 (12)	
	**Education, n** **(%)**			
		High school or less	15 (15)	
		Some college	47 (47)	
		BA^a^, BS^b^, or higher	38 (38)	
**Smoking characteristics**				
	Number of days smoked in past 30 days, mean (SD)			14.7 (4.6)
	Number of cigs smoked per smoking day, mean (SD)			4.6 (3.3)
	Ever smoked daily? (yes), n (%)			70 (70)
	Ever quit before? (yes), n (%)			77 (77)

^a^BA: Bachelor of Arts.

^b^BS: Bachelor of Science.

### Feasibility as Captured by App Use

Participants used the SiS2 app on average on 24.1 (SD 14.1, range 0-49) days out of the 49 prescribed days after onboarding (49% of days). Most participants used the app on their own on the day after their onboarding call (96/100, 96%). During the initial week of app use (ie, the week leading up to the chosen quit day), the percentage of participants interacting with the app on a given day decreased to 60% (60/100). This decrease was largely because of participants settling into a less-than-daily routine of using the app rather than participants discontinuing app use altogether: 7% (7/100) used the app on 0-1 days after onboarding during the first week, 53% (53/100) on 2-5 days, and 40% (40/100) on 6-7 days. App use declined further at a gradual pace during the remaining 6 weeks of prescribed app use, so that during the last week, 33% (33/100) used the app on 0 days, 12% (12/100) on 1 day, 35% (35/100) on 2-5 days, and 20% (20/100) on 6-7 days. More than a quarter of the participants (28/100, 28%) used the SiS2 app at least three times per week for every week of treatment.

### Acceptability as Captured by the End of the Treatment (Week 6) Survey Responses

The average score on the SUS, as measured at the end of treatment, was 79.8 (SD 17.3), which represents an *A* grade [48]. Participants rated the specific functions of the app (Table 2) as *easy* to *very easy* to use. These ratings ranged from 2.3 (SD 0.9) for playing *Magma Bear* to 2.7 (SD 0.6) for viewing earned badges. Essential features, including completing the positive psychology exercises, completing the behavioral challenges, and using the cigarette log, all scored high and in this range, with 62% (59/95), 64% (61/95), and 74% (70/95), indicating that these functions, respectively, were *very easy to use*.

**Table 2:** User ratings of the Smiling Instead of Smoking app version 2 functions (N=95).

User ratings		Ease of use^a^		Useful^b^	
		Value, mean (SD)	Very easy, n (%)	Value, mean (SD)	Very useful, n (%)
**System Usability Scale**					
	SUS^c^ score^d^	79.8 (17.3)	—^e^	—	—
**Happiness related tasks**					
	Completing the positive psychology exercises every day	2.4 (0.9)	59 (62)	2.2 (0.9)	44 (47)
	Specifically, completing *3 Good Things*	2.4 (0.9)	55 (58)	2.2 (0.9)	48 (51)
	Specifically, completing *Savoring*	2.4 (0.8)	56 (59)	2.2 (0.9)	45 (47)
	Specifically, completing *Experiencing Kindness*	2.4 (0.9)	58 (62)	2.1 (1.0)	45 (47)
	Specifically, completing *Reliving Happy Moments*	2.5 (0.8)	64 (67)	2.2 (0.9)	47 (49)
	Specifically, completing *Rose, Thorn, and Bud*	2.4 (0.8)	56 (59)	2.1 (1.0)	43 (45)
	Viewing the Happiness Log of past exercise completions.	2.6 (0.7)	70 (74)	2.2 (0.9)	42 (44)
	Viewing Owl Wisdoms (ie, happiness science findings)	2.6 (0.6)	61 (64)	2.2 (0.9)	45 (47)
**Smoking specific tasks**					
	Setting (and if applicable resetting) the quit day	2.5 (0.8)	62 (65)	2.1 (1.0)	42 (44)
	Completing the behavioral challenges	2.5 (0.7)	61 (64)	2.2 (0.9)	46 (48)
	Accessing and updating the cigarette log	2.6 (0.7)	70 (74)	2.4 (0.8)	53 (56)
	Using the *Magma Bear* game	2.3 (0.9)	54 (57)	1.3 (1.2)	26 (27)
	Setting and receiving *Smoke Alarms*	2.4 (0.8)	53 (56)	2.1 (1.0)	42 (44)
	Specifying personal reasons for quitting smoking	2.5 (0.8)	59 (62)	2.3 (0.8)	51 (54)
	Viewing strategies for remaining smoke-free	2.5 (0.7)	60 (63)	2.3 (0.9)	49 (52)
	Viewing earned badges	2.7 (0.6)	66 (69)	2.0 (1.0)	40 (43)
	Viewing benefits of quitting smoking	2.6 (0.6)	61 (64)	2.2 (0.9)	45 (48)

^a^Ease of use was rated on a 4-point scale: 0=not easy at all, 1=somewhat easy to use, 2=easy to use, 3=very easy to use.

^b^Usefulness was rated on a 4-point scale: 0=not at all useful, 1=somewhat useful, 2=useful, 3=very useful.

^c^SUS: System Usability Scale; scores can range from 0 (very poor perceived usability) to 100 (excellent perceived usability) in 2.5-point increments.

^d^A+=84.1-100; A=80.3-84.0; B=68-80.3.

^e^The System Usability Scale (SUS) presents a general usability score (does not differentiate between ease of use and usefulness) and uses a different rating scale (1=strongly disagree, 5=strongly agree) than the app’s ease of use and usefulness scale (ease of use was rated as 0=not easy at all, 1=somewhat easy to use, 2=easy to use, 3=very easy to use, and usefulness was rated as 0=not at all useful, 1=somewhat useful, 2=useful, 3=very useful). For this reason, SUS values for ease of use (very easy) and useful (mean, very useful) were not included.

Greater variations existed in the perceived usefulness of the functions. These ratings ranged from 1.3 (SD 1.2) for playing *Magma Bear* to 2.4 (SD 0.8) for using the cigarette log. Other than *Magma Bear*, all functions scored as at least *useful* or higher on average. In particular, high-scoring functions were the cigarette log (53/95, 56% of participants rated it as *very useful*), viewing strategies for remaining smoke-free (49/95, 52%), and the happiness exercise *Reliving Happy Moments* (47/95, 49%).

In rating how the SiS2 app might have helped them to quit smoking (Table 3), nearly all participants (87/95, 92% of participants who responded to this item) indicated that the SiS2 app served as a useful reminder of why quitting smoking was important to them. The next most useful app aspect, by participant ratings, was that the SiS2 app showed them how happiness was important in numerous ways (82/95, 86% of participants). Organizational and teaching goals were also met by the SiS2 app, with participants indicating that the SiS2 app helped them stay on track (80/95, 84% of participants), helped them prepare for the quit attempt (80/95, 84% of participants), and provided confidence in the steps to take (78/95, 82% of participants). For some smokers, the SiS2 app was useful in dealing with specific risky smoking times that arose during this quit attempt (65/95, 68% of participants). Overall, most participants felt that the SiS2 app helped them in their quit attempt (83/95, 87% of participants) and that most would recommend it to a friend (87/95, 92% of participants).

**Table 3:** Ratings of how the Smiling Instead of Smoking app version 2 might have helped (N=95).

User ratings^a^			Value, mean (SD)		Agree and Strongly agree, n (%)
**The SiS^b^app...**					
	...reminded me why I wanted to quit.	4.4 (0.9)		87 (92)	
	...reminded me that quitting was important to me.	4.4 (0.8)		87 (92)	
	...showed me how happiness is important in numerous ways.	4.3 (0.9)		82 (86)	
	...made me think that it was worthwhile for me to quit.	4.3 (0.9)		81 (85)	
	...helped remind me to stay on track with quitting.	4.2 (1.0)		80 (84)	
	...helped me prepare for the quit attempt.	4.3 (1.0)		80 (84)	
	...helped me stay positive while quitting.	4.2 (1.0)		78 (82)	
	...gave me a sense of accomplishment as I progressed through my quit attempt.	4.3 (0.9)		78 (82)	
	...made me feel that I knew the right steps to take to quit.	4.2 (0.9)		78 (82)	
	...made me take my quit attempt seriously.	4.2 (1.0)		76 (80)	
	...gave me confidence that I can quit smoking.	4.0 (0.9)		73 (77)	
	...reminded me in crucial moments to stay quit.	4.1 (1.0)		72 (76)	
	...made me feel that someone cared if I quit.	4.1 (1.0)		70 (74)	
	...encouraged me when things were getting tough.	4.0 (1.0)		70 (74)	
	...gave me the feeling I could get trusted advice at any time.	3.9 (1.1)		67 (71)	
	...helped me deal with risky smoking times.	3.9 (1.1)		65 (68)	
	...gave me a new toy to play with rather than dwell on quitting.	3.7 (1.3)		60 (63)	
**Overall ratings**					
	Would you recommend the SiS app to a friend who wants to quit smoking? (yes)	—^c^		87 (92)	
	Taken altogether, do you think that the app helped you in your quit attempt? (yes)	—		83 (87)	

^a^Rated on a 5-point Likert scale: 1=strongly disagree, 2=disagree, 3=neither agree nor disagree, 4=agree, and 5=strongly agree.

^b^SiS: Smiling Instead of Smoking.

^c^The overall rating questions were answered as yes or no, therefore, there is no mean for this item.

### Tests of Within-Person Changes Predicted by the SiS2 Conceptual Model

Most, but not all, of the hypothesized within-person changes were observed from baseline to end of treatment (Table 4). In line with our conceptual model, the results indicated that the self-efficacy for remaining abstinent significantly increased for both internal cues (*P*<.001) and external cues (*P*<.001). Furthermore, participants’ desire to smoke decreased (*P*<.001) and perceptions of smoking became less positive, as expressed through reduced valuing of psychoactive benefits and pleasure of smoking, as well as reductions in the perceived importance of the pros of smoking (Table 4). Effects were large for decreases in desire to smoke, importance of the pros of smoking, and ratings of the psychoactive benefits of smoking (ie, all Hedges g_av_≥0.80), and effects were of medium size for self-efficacy and the pleasure of smoking (ie, all Hedges g_av_≥0.49).

**Table 4:** Within-person changes on theorized mechanisms of change from baseline to end of treatment.

Construct (scale)				Cronbach α at baseline^a^		Baseline^b^, mean (SD)		Scale range		6-week^c^ versus baseline				
										b^d^ (95% CI)		*P* value		g_av_
**Self-efficacy**														
	SEQ-12^e^ (internal cues)		.86		53.4 (22.0)		0-100		13.1 (7.6 to 18.7)		<.001		0.53	
	SEQ-12 (external cues)		.77		58.9 (21.1)		0-100		11.1 (6.1 to 16.1)		<.001		0.49	
**Desire to smoke**														
	QSU^f^ (smoking urges)		.92		3.7 (1.4)		1-7		−1.5 (−1.9 to −1.1)		<.001		1.01	
**Processing self-relevant health information**														
	**Positive appraisals of smoking**													
		ATS^g^ (psychoactive benefits)	.77		4.0 (0.7)		1-5		−0.8 (−1.0 to −0.5)		<.001		0.80	
		ATS (pleasure)	.84		3.3 (1.0)		1-5		−0.6 (−0.8 to −0.3)		<.001		0.52	
		DCB^h^ (importance of the pros of smoking)	.67		56.6 (19.9)		0-100		−20.7 (−27.2 to −14.3)		<.001		0.83	
	**Negative appraisals of smoking**													
		ATS (adverse effects)	.84		4.4 (0.5)		1-5		0.0 (−0.1 to 0.2)		.76		0.03	
		DCB (importance of cons of smoking)	.80		68.6 (26.2)		0-100		−2.9 (−8.1 to 2.3)		.27		0.09	
	**Benefits and barriers to quitting smoking**													
		Single item (pros of quitting)	N/A^i^		84.9 (21.1)		0-100		−9.1 (−15.9 to −2.3)		.009		0.35	
		Single item (cons of quitting)	N/A		63.4 (32.3)		0-100		−5.1 (−13.7 to 3.6)		.25		0.14	
	**Motivation to quit smoking**													
		CQSS^j^ (commitment to quitting)	.89		4.1 (0.7)		1-5		−0.1 (−0.3 to 0.1)		.25		0.09	
		Single item (how motivated)	N/A		88.0 (14.6)		0-100		−6.6 (−11.1 to −2.0)		.005		0.34	
**Positive affect**														
	PANAS^k^ (past week positive affect)		.66		3.0 (0.6)		1-5		0.0 (−0.1 to 0.2)		.45		0.08	
	PANAS (past week negative affect)		.72		2.7 (0.7)		1-5		0.0 (−0.1 to 0.2)		.74		0.02	
	Single item (how happy past week)		N/A		67.7 (21.5)		0-100		1.2 (−3.8 to 6.3)		.62		0.09	
	Single item (how happy right now)		N/A		70.0 (20.4)		0-100		−0.5 (−5.4 to 4.4)		.83		0.00	
	Satisfaction with life		.87		4.4 (1.4)		1-7		0.3 (−0.1 to 0.6)		.11		0.18	
	Subjective happiness		.87		4.8 (1.4)		1-7		0.2 (−0.1 to 0.4)		.16		0.12	

^a^Cronbach α is a measure of the internal consistency of each scale at baseline.

^b^Baseline occurred before Smiling Instead of Smoking app download.

^c^6-week follow-up occurred at the end of the prescribed 49 days of app use (ie, 6 weeks post quit day).

^d^b is the parameter estimate of the pairwise difference of week 6 compared with baseline from the repeated measures mixed effects model.

^e^SEQ-12: Smoking Self-Efficacy Questionnaire.

^f^QSU: Brief Questionnaire of Smoking Urges.

^g^ATS: Attitudes Towards Smoking Scale.

^h^DCB: Decisional Balance Inventory for Smoking.

^i^N/A: not applicable (Cronbach α is not applicable to single-item measures).

^j^CQSS: Commitment to Quitting Smoking Scale.

^k^PANAS: Positive and Negative Affect Schedule.

Contrary to expectations, motivation to quit smoking and perceived importance of the pros of quitting decreased below baseline levels by the end of treatment (*P*=.005 and *P*=.009, respectively). Both effects were smaller than the other observed effects (ie, Hedges g_av_≤0.35). Post hoc analyses showed that motivation to quit decreased from baseline to week 6 for those who did not succeed in quitting (*b*=−14.7, 95% CI −20.1 to −9.2; *P*<.001), whereas it tended to increase for those who did (*b*=5.0, 95% CI −1.5 to 11.4; *P*=.13; interaction *P*<.001). No such interaction effect existed for the perceived importance of pros of quitting (abstinent: *b*=−9.5, 95% CI −20.0 to 1.1; *P*=.08; nonabstinent: *b*=−8.9, 95% CI –17.8 to 0.0; *P*=.05; interaction *P*=.61). We were unable to detect any changes in the negative appraisals of smoking, cons of quitting, or commitments to quitting. Of note, the perceptions of the adverse effects of smoking (ie, Attitudes Towards Smoking Adverse Effects subscale) were already very high at baseline, with an average score of 4.4 (SD 0.5) out of 5 (Table 4), indicating the possibility of a ceiling effect for this measure. However, in terms of the importance of these *cons* of smoking, there was some room for improvement, with an average baseline level of 68.8 (SD 26.2) on a scale of 0-100. The results of the exploratory analyses of changes in positive affect are included in Table 4. In line with our expectations, we were unable to detect any changes in positive affect while smokers underwent the quit attempt.

### Self-reported Smoking Cessation Rates

The self-reported 30-day point prevalence abstinence rates at the end of treatment (ie, 6 weeks after the quit date) was 40% (40/100 participants; participants who did not complete the survey were presumed to be smoking). Self-reported abstinence increased further during follow-up, with 30-day point prevalence abstinence reported by 56% (56/100) of participants at both 12 and 24 weeks after the quit day. Participants who did not complete the surveys were assumed to be smoking.

## Discussion

### Principal Findings

We tested version 2 of the SiS app (SiS2) in a single-group longitudinal study using minimal contact and no face-to-face contact. We found that the SiS2 app was a feasible and acceptable mHealth tool to support nondaily smokers in quitting smoking.

Nondaily smokers were engaged well by the SiS2 app (67/100, 67% of participants still used the app in the seventh week after enrollment) and found it useful. App use is an important outcome metric as it is through interaction with an app that app users are engaged in therapeutic activities. Achieving high app use is currently a critical barrier that challenges the utility of publicly available apps, and a recent study on mental health apps found that <4% of app users used the app 15 days after installation in real-world settings [60]. In clinical trials, app use is expected to be higher for a number of reasons (eg, actively procuring commitment from study participants to study procedures, which they may interpret to include app adherence) [61] but is nevertheless a pivotal marker of feasibility. In our study, participants used the SiS2 app for an average of 24.7 days. To put this app use into context, it may be useful to contrast it with app use observed in a recent randomized controlled trial [62], which represents the current best evidence regarding smoking cessation apps. In this study, Bricker et al [62] used a double-blind, individually randomized, 2-group stratified design to test the efficacy of their app, called iCanQuit, which is based on acceptance and commitment therapy. They compared it with the publicly available smartphone app QuitGuide, which was developed and disseminated by the National Cancer Institute (NCI). In this study, participants used the QuitGuide app on 7.1 days, on average, and the iCanQuit app on 24.3 days. Thus, the app use of 24.7 days observed for the SiS2 app is in line with the best standard set to date by the iCanQuit app. However, in our study, participants engaged in a phone call with study staff to get oriented to the app, in line with how we conceptualized such an app should be used in a health care setting, which was not the case for the Bricker et al [62] study, in which participants only received a link to download and install the app [62]. We also told participants at baseline that we would like to conduct a Skype (Microsoft Corporation) interview with a subsample of the study participants (20/100, 20%) at the end of the treatment about their experience using the app and asked them if they would like to be considered for such an interview (US $25 was offered for the Skype interview; 95/100, 95% said yes). Although it is standard practice for this type of treatment evaluation research to include an exit interview, this question may have also created motivation for greater app use than may happen in a real-world setting, although notably, following up with patients after a smoking cessation referral is part of best practice guidelines (ie, the *arrange follow-up* portion of the 5As) [63]. In line with the *law of attrition* for digital technologies [64], there was app use attrition after an initial curiosity phase; however, there was also sustained use of the app throughout the 7 weeks of the prescribed app use period, and more than a quarter of our sample (28/100, 28%) engaged meaningfully with the SiS2 app every week of the 7-week treatment period. This finding was particularly encouraging, given that we had increased the prescribed app use period from 3 to 7 weeks in going from version 1 to version 2 of the app.

In terms of the perceived usefulness of the app, our data further suggest that we were able to improve perceptions of the usefulness of the positive psychology content of the SiS app. In comparing usefulness ratings across the SiS1 and SiS2 studies, we noted that the perceived usefulness of *completing the positive psychology exercises every day* increased from 1.8 in study 1 [24] to 2.2 in study 2. It is difficult, of course, to draw direct conclusions from this improvement in scores as the demographics of the samples were also quite different (ie, largely Black males in SiS1 vs White females in SiS2). Overall, 87% (83/95) of the nondaily smokers participating in our study felt that using the SiS2 app helped them in their quit attempt. This compares favorably with the usefulness ratings of 80% for the iCanQuit app and 72% for the NCI QuitGuide app [62]. Similarly, in terms of recommending the app to a friend, SiS2 fared well (87/95, 92% for SiS2 compared with 83% and 71% for the iCanQuit and QuitGuide apps, respectively) [62]. Together, these findings demonstrate high levels of engagement and positive perceptions of the usefulness of the SiS2 app.

In terms of the user interface, SiS2 scored well (*A* grade, 79.8) but left room for improvement. To put this score into context, it may be useful to consider 2 other studies that have used the SUS scale to evaluate smoking cessation apps. In an early stage study of user experiences of smokers with serious mental illness (n=5), the NCI smartphone app *QuitPal* received a SUS score of 66 [65]. In a larger trial in the same study population, participants were randomized to use the NCI app QuiteGuide or the investigator developed app *Learn to Quit* [66]. In this study, SUS scores were 78 and 85, respectively, for the apps QuitGuide and *Learn to Quit.* In these 2 studies, onboarding was performed in person and included additional help over time, as needed. In our case, onboarding was performed remotely in a single phone call. Thus, our robust SUS score suggests that the SiS2 app is implementable via low- to no-touch linkage approaches. Our data further suggest that the change from weekly smoking cessation modules to shorter, more frequent *behavioral challenges* improved the ease of use of the app. In comparing the SiS1 and SiS2 ease of use scores for these features of the apps, we saw a noticeable increase: from a score of 2.0 (*easy to use*) for *completing the smoking sessions* [24] to 2.5 (midpoint between *easy to use* and *very easy to use*) for *completing the behavioral challenges*. This suggests that it may indeed be useful to break larger asks within an app into smaller, simpler asks, so long as they are meaningful.

Findings from the within-person tests were largely in line with our conceptual model. Overall perceptions regarding smoking changed as expected: participants reported greater confidence to abstain from smoking when faced with internal or external stimuli, experienced a lower desire to smoke, and decreased their positive appraisal of smoking over time. Notable exceptions to our expected within-person changes were the unexpected decreases in the single-item measures capturing motivation to quit smoking and the importance of the pros of quitting. Our post hoc analyses provide some insight into possible explanations for the decrease in motivation, where it appeared that motivation to quit smoking was negatively affected by having tried and failed, in line with the abstinence violation effect [67]. The decrease in the perceived importance of the pros of quitting smoking suggests that as smokers enter the maintenance phase of smoking cessation, the pros of quitting have decreased salience. As these scores at this point in time do not relate to smoking cessation success prospectively, this finding suggests that it may not be fruitful to emphasize the pros of quitting at this point of the smoking cessation process in mHealth tools designed to support quitting smoking and maintaining smoking abstinence. By and large, the tests of within-person changes are encouraging in that they suggest that the hoped-for cognitive and emotional changes are indeed taking place as nondaily smokers engage with the SiS2 app. Indeed, interactions with other mHealth smoking cessation technologies have produced similar results [68], although with somewhat weaker effects. Further testing of the SiS2 app in a randomized design is warranted to test the degree to which these changes are attributable to SiS2 app use.

The self-reported 30-day point prevalence abstinence rates we observed are very promising (eg, 56% abstinent at the 6-month follow-up) and certainly exceed expectations for this type of technology. Expected smoking abstinence rates (for daily smokers) are 20%-25% (treatment) versus 14%-16% (control) for SMS text messaging [69,70], and 28% (treatment) versus 21% (control) for an app [62]. However, it must be kept in mind that our study was a single-group longitudinal study, with a strong potential for a response bias, given that there was no blinding and that participants were onboarded by a motivated study staff member. Nevertheless, these high self-reported 30-day abstinence rates indicate that the SiS2 app merits study in a randomized trial.

### Limitations

This was a single-group longitudinal study without biochemical verification of smoking status. There is the potential for a response bias in reporting smoking status and potentially regarding our other self-report measures as well. We reminded participants during all stages of the study (ie, during screening, during enrollment, and during survey taking) that their honest reporting was of critical importance to us, even and especially if they did not like something about the app. Note also that our primary feasibility outcome indicator, actual app use, was not subject to self-report biases, as it was automatically recorded as it occurred. Second, our study approach used an interactive onboarding procedure, done via phone, where a staff member was guiding participants through downloading, installing, and using the app. Thus, our results may not generalize to referral-only situations, where smokers find the app on their own or are merely referred to it. We used the onboarding call as it builds on best practices for warm linkages to community resources, similar to a warm handoff for linking hospitalized smokers to quitlines [71], which may be particularly helpful for stigmatized and underserved populations [72]. In addition, our eligibility criteria included willingness to make a quit attempt as part of the study. Motivation is an essential factor in smoking cessation. By restricting study participation to those who are willing to make a quit attempt, this study likely restricted participation to those with a relatively high level of motivation to quit smoking. This focus reflects real-world settings, as individuals do not access smoking cessation programs unless they are motivated. Indeed, national quitlines require that smokers be motivated to quit to use their services. With regard to apps, those not willing to make a quit attempt are unlikely to go to the app store to find a smoking cessation app. The SiS app is designed for those smokers who want to quit smoking and who want to use an app to help them do so. Third, our sample was predominantly White (75/100, 75%), unlike our previous sample in study 1, where Black nondaily smokers were the largest racial group (43%) [24]. Future analyses will examine in depth whether any demographic or clinical characteristics predicted more or less engagement with the SiS2 app.

### Conclusions

The SiS2 app was feasible, acceptable, showed promising changes in constructs relevant to smoking cessation, and had high self-reported quit rates by nondaily smokers. The SiS2 app warrants testing in a randomized controlled trial.

## Abbreviations

mHealth: mobile health
NCI: National Cancer Institute
NRT: nicotine replacement therapy
REDCap: Research Electronic Data Capture
SiS: Smiling Instead of Smoking
SUS: System Usability Scale
